# The Structure-Function Relationship of Angular Estrogens and Estrogen Receptor Alpha to Initiate Estrogen-Induced Apoptosis in Breast Cancer Cells[Fn FN3]

**DOI:** 10.1124/mol.120.119776

**Published:** 2020-07

**Authors:** Philipp Y. Maximov, Balkees Abderrahman, Yousef M. Hawsawi, Yue Chen, Charles E. Foulds, Antrix Jain, Anna Malovannaya, Ping Fan, Ramona F. Curpan, Ross Han, Sean W. Fanning, Bradley M. Broom, Daniela M. Quintana Rincon, Jeffery A. Greenland, Geoffrey L. Greene, V. Craig Jordan

**Affiliations:** Departments of Breast Medical Oncology (P.Y.M., B.A., P.F., D.M.Q.R., J.A.G., V.C.J.) and Computational Biology and Bioinformatics (B.M.B.), University of Texas, MD Anderson Cancer Center, Houston, Texas; King Faisal Specialist Hospital and Research (Gen.Org.), Research Center, Jeddah, Kingdom of Saudi Arabia (Y.M.H.); The Ben May Department for Cancer Research, University of Chicago, Chicago, Illinois (R.H., S.W.F., G.L.G.); Center for Precision Environmental Health and Department of Molecular and Cellular Biology (C.E.F.), Mass Spectrometry Proteomics Core (A.J., A.M.), Verna and Marrs McLean Department of Biochemistry and Molecular Biology, Mass Spectrometry Proteomics Core (A.M.), and Dan L. Duncan Comprehensive Cancer Center (A.M., C.E.F.), Baylor College of Medicine, Houston, Texas; Adrienne Helis Malvin Medical Research Foundation, New Orleans, Louisiana (Y.C.); and Coriolan Dragulescu Institute of Chemistry, Romanian Academy, Timisoara, Romania (R.F.C.)

## Abstract

High-dose synthetic estrogen therapy was the standard treatment of advanced breast cancer for three decades until the discovery of tamoxifen. A range of substituted triphenylethylene synthetic estrogens and diethylstilbestrol were used. It is now known that low doses of estrogens can cause apoptosis in long-term estrogen deprived (LTED) breast cancer cells resistant to antiestrogens. This action of estrogen can explain the reduced breast cancer incidence in postmenopausal women over 60 who are taking conjugated equine estrogens and the beneficial effect of low-dose estrogen treatment of patients with acquired aromatase inhibitor resistance in clinical trials. To decipher the molecular mechanism of estrogens at the estrogen receptor (ER) complex by different types of estrogens—planar [17*β*-estradiol (E_2_)] and angular triphenylethylene (TPE) derivatives—we have synthesized a small series of compounds with either no substitutions on the TPE phenyl ring containing the antiestrogenic side chain of endoxifen or a free hydroxyl. In the first week of treatment with E_2_ the LTED cells undergo apoptosis completely. By contrast, the test TPE derivatives act as antiestrogens with a free para-hydroxyl on the phenyl ring that contains an antiestrogenic side chain in endoxifen. This inhibits early E_2_-induced apoptosis if a free hydroxyl is present. No substitution at the site occupied by the antiestrogenic side chain of endoxifen results in early apoptosis similar to planar E_2_. The TPE compounds recruit coregulators to the ER differentially and predictably, leading to delayed apoptosis in these cells.

**SIGNIFICANCE STATEMENT:**

In this paper we investigate the role of the structure-function relationship of a panel of synthetic triphenylethylene (TPE) derivatives and a novel mechanism of estrogen-induced cell death in breast cancer, which is now clinically relevant. Our study indicates that these TPE derivatives, depending on the positioning of the hydroxyl groups, induce various conformations of the estrogen receptor’s ligand-binding domain, which in turn produces differential recruitment of coregulators and subsequently different apoptotic effects on the antiestrogen-resistant breast cancer cells.

## Introduction

Breast cancer has the highest incidence of all cancers in women in the United States with more than 200,000 new cases diagnosed each year and almost 40,000 deaths in 2015 (Siegel et al., 2015). It is estimated that the number of newly diagnosed cases will considerably increase in the next 15 years and that the great majority of breast cancer cases will be estrogen receptor (ER) alpha positive (Anderson et al., 2011). As a result, it is essential to understand the vulnerabilities of ER-positive breast cancer so new treatment strategies can be devised.

High-dose estrogen therapy was the standard therapy for advanced breast cancer for three decades (Haddow et al., 1944; Kennedy, 1965) until the discovery of tamoxifen (Jordan, 2003). However, estrogen therapy was most effective in patients at least 5 years past their menopause (Haddow, 1970). The reason for this observation was unknown. Synthetic estrogens such as diethylstilbestrol (DES) and triphenylethylene (TPE) derivatives were tested for their therapeutic efficacy (Haddow et al., 1944). Only DES was used as the more effective agent, despite the fact that it had more systemic side effects than the TPE derivatives (Kennedy, 1965). Years later (Jordan et al., 2001), synthetic estrogens were classified into two different estrogen types: class I [planar compounds like 17*β*-estradiol (E_2_) and DES] and class II [angular (TPEs)] estrogens. The classification was based on the efficacy of planar estrogen–ER complexes to activate an estrogen target gene in stably transfected target cells, which an angular TPE estrogen did not do. All estrogens are not the same.

Human breast cancer models in vivo and in vitro that acquire antiestrogen (tamoxifen) resistance or experience long-term estrogen deprivation expose (Wolf and Jordan, 1993; Yao et al., 2000) a vulnerability for low-dose estrogen–induced apoptosis (Song et al., 2001; Lewis et al., 2005a; Ariazi et al., 2011). In earlier studies, the evolution of breast cancer cell resistance in vivo was described; short-term antiestrogen therapy (1 to 2 years) caused the ER-positive breast cancer cells to grow robustly with tamoxifen (Gottardis and Jordan, 1988; Gottardis et al., 1989); however, 5 years of estrogen deprivation with tamoxifen created a cell phenotype in which cells have enhanced growth rate, but treatment of transplanted animals with low-dose E_2_ induced apoptosis (Yao et al., 2000). Most importantly, the 5 years of estrogen deprivation in laboratory is reminiscent of Haddow’s clinical observation (Haddow, 1970) that women must be 5 years past menopause for estrogen therapy to be effective to treat breast cancer in postmenopausal women. These experimental findings have clinical parallels today.

Clinical data from the Women’s Health Initiative estrogen-alone trial demonstrate that estrogens are able to reduce the incidence of breast cancer in postmenopausal women over the age of 60, even after the termination of estrogen therapy (Anderson et al., 2004; LaCroix et al., 2011; Chlebowski et al., 2019). Estrogen therapy also has clinical benefit in metastatic breast cancer with antihormone resistance (Lønning et al., 2001; Ellis et al., 2009; Schmidt et al., 2019). The clinical relevance of estrogen-induced apoptosis justifies the study of molecular mechanisms of the estrogen-ER complex in appropriate preclinical models to explore further clinical applications of this translational research knowledge.

**Fig. 1.**
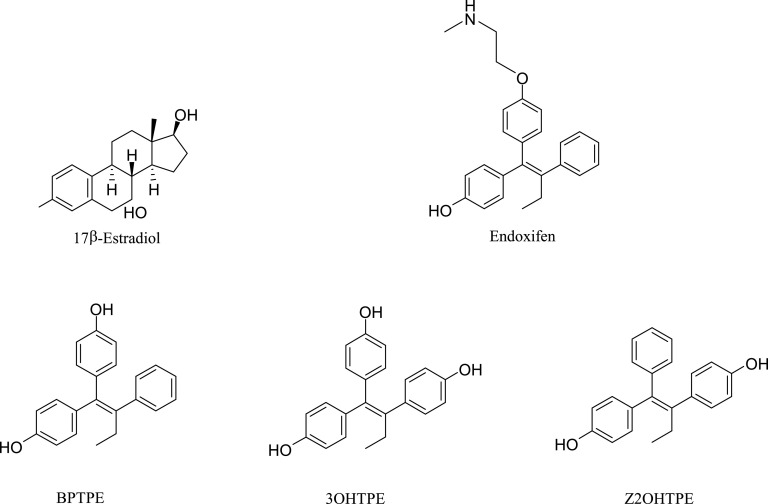
Chemical structures of compounds used in this study. The synthesis of the compounds BPTPE, 3OHTPE, and Z2OHTPE was described previously (Maximov et al., 2010).

Previously, we have shown that ethoxytriphenylethylene (EtOXTPE) induced a novel conformation of the ligand-binding domain (LBD) of the ER as resolved by X-ray crystallography (Maximov et al., 2018). Although EtOXTPE was a mixture of geometric isomers, only the Z-isomer crystallized in the ER complex. In this paper we employ a panel of TPE derivatives of precise structure (Fig. 1), which include bisphenoltriphenyltheylene (BPTPE), trihydroxytriphenylethylene (3OHTPE), and *trans*-isomer of dihydroxytriphenylethylene (Z2OHTPE). Here we demonstrate that the structure-function relationship of the ER bound with different TPE derivatives creates unique three-dimensional ER conformations that affect the binding of distinct subsets of coregulators. The structure-function relationships correlate with the molecular events over time that cause estrogen-induced apoptosis in long-term estrogen deprived (LTED) breast cancer cells.

## Experimental Procedures

### 

#### Reagents and Cell Culture.

E_2_ was purchased from Sigma-Aldrich (St. Louis, MO). Endoxifen was purchased from Santa Cruz Biotechnology (Dallas, TX). Z2OHTPE, 3OHTPE, and BPTPE (Fig. 1) were synthesized and structures characterized at the Fox Chase Cancer Center Organic Synthesis Facility, Philadelphia, PA, as previously described (Maximov et al., 2010). All compounds were dissolved in ethanol and were stored at −20°C and protected from light. MCF-7:5C cells were maintained in phenol-red–free RPMI 1640 supplemented with 10% charcoal stripped fetal serum, 2 mM glutamine, penicillin at 100 U/ml, streptomycin at 100 μg/ml, 1× nonessential amino acids, and bovine insulin at 6 ng/ml. Cells were cultured in T75 culture flasks (Thermo Scientific, Pittsburgh, PA) and passaged twice a week at 1:3 ratio. All cultures were grown in 5% CO_2_ at 37°C.

#### Cell Proliferation Assays.

All pharmacological properties of the investigated compounds were evaluated by assessing the cell proliferation of the ER-positive MCF-7:5C cells by measuring the amount of DNA from the cells after treatments. Cells were seeded into 24-well plates at a density of 10,000 cells per well for 1-week treatment or 5000 cells per well for a 2-week treatment in MCF-7:5C cells. The next day, cells were treated with culture medium containing the test compounds. The medium was changed every 2 days for the whole duration of the experiment. All treatments were performed in triplicate. On the last day of the treatments cells were harvested by medium aspiration and washed in ice-cold PBS (Life Technologies, Carlsbad, CA) once and analyzed using the DNA quantification kit (Bio-Rad, Hercules, CA) according to the manufacturer’s instructions. Samples were quantitated on a Synergy H1 plate reader (BioTek Instruments Inc., Winooski, VT) in black wall 96-well plates (Nalge Nunc International, Rochester, NY). All growth assays were performed in triplicate, the results represent the average of all replicates, and the error bars represent the S.D. in each treatment. Key differences are described in the *Results* section with 95% confidence intervals (CIs).

#### Annexin V Staining.

MCF-7:5C cells were seeded at 300,000 cells per 10-cm Petri dish for 6-day treatments and 700,000 cells for 3-day treatment. Cells were treated the next day with test compounds for 6 days and for 3 days with 1 nM E_2_. Cells were harvested by aspirating media and washing cells with warm PBS twice and subsequently treated with Accutase solution (Life Technologies, Grand Island, NY) for 4 minutes at 37°C. Cells were then harvested by pipetting after addition of PBS and then transferred to centrifuge tubes and centrifuged. Cells were put on ice afterward and stained using FITC Annexin V Apoptosis Detection Kit I (BD Pharmingen, San Diego, CA) according to the manufacturer’s instructions. The samples were read using BD Accuri C6 Plus flow cytometer (Becton, Dickinson and Company, Franklin Lakes, NJ). The assay was performed in triplicate; data shown represent one of the biologic replicates. The total percentages of apoptotic cells were quantified after the addition of the numbers of Annexin V–positive cells labeled as apoptotic and the Annexin V/propidium iodide double positive cells labeled as dead cells.

#### Real-Time Polymerase Chain Reaction.

Cells were seeded the day prior to treatment in 24-well plates at a density of 100,000 cells per well. After the indicated durations of treatments, the cells were harvested, and RNA was isolated using MagMAX-96 Total RNA Isolation Kit (Applied Biosystems, Carlsbad, CA) and processed using Kingfisher Duo Prime magnetic particle processor (Thermo Scientific, Waltham, MA) according to the manufacturer’s instructions. Subsequently cDNA was synthesized using High Capacity cDNA Reverse transcription kit (Applied Bioscience, Carlsbad, CA) according to the manufacturer’s instructions using 1 µg of purified RNA. Synthesized cDNA was diluted in nuclease-free water and used for real-time polymerase chain reaction (PCR). For real-time PCR a Power SYBR green PCR master mix was used (Applied Bioscience) according to the manufacturer’s instructions. Real-time PCR was performed using a QuantStudio 6 Flex Real Time PCR thermocycler (Applied Bioscience). All primers were obtained from Integrated DNA Technologies Inc. (Coralville, IA) and were validated by melt curve analysis that revealed single peaks for all primer pairs. Primers sequences that were used for human *TFF1* cDNA amplification are 5′-CAT​CGA​CGT​CCC​TCC​AGA​AGA-3′ sense and 5′-CTC​TGG​GAC​TAA​TCA​CCG​TGC​TG-3′ antisense; human *GREB1* gene: 5′-CAA​AGA​ATA​ACC​TGT​TGG​CCC​TGC-3′ sense and 5′-GAC​ATG​CCT​GCG​CTC​TCA​TAC​TTA-3′ antisense; human *BCL2L11* gene: 5′-TCG​GAC​TGA​GAA​ACG​CAA​G-3′ sense and 5′-CTC​GGT​CAC​ACT​CAG​AAC​TTA​C-3′ antisense; human *TP63* gene: 5′-TTC​GGA​CAG​TAC​AAA​GAA​CGG-3′ sense and 5′-GCA​TTT​CAT​AAG​TCT​CAC​GGC-3′ antisense; and the reference gene *RPLP0*, 5′-GTG​TCC​GAC​AAT​GGC​AGC​AT-3′ sense and 5′-GAC​ACC​CTC​CAG​GAA​GCG​A-3′ antisense. The fold changes of the mRNA after treatments with test compounds versus vehicle controls were calculated using ΔΔCt method. All treatments were performed in triplicate, the results represent the average of all replicates, and the error bars represent the S.D. in each treatment. Key differences are described in the *Results* section with 95% CIs.

#### Chromatin Immunoprecipitation.

Assays were performed on MCF-7:5C cells grown in 15-cm Petri dishes to approximately 80% confluency. The cells were treated for 45 minutes in full growth media with the tested compounds after which the cells were washed once with warm PBS and then crosslinked with 1% formaldehyde in PBS for 10 minutes. The crosslinking reactions were quenched with 0.125 M glycine and subsequently washed twice with ice-cold PBS. Cells were collected by scraping and collected into PBS with Halt protease and phosphatase inhibitor cocktail (Thermo Fisher Scientific). Cells were pelleted by centrifugation, and chromatin was isolated using Pierce Magnetic ChIP kit (Thermo Fisher Scientific) according to manufacturer’s instructions. Antibodies used for the immunoprecipitations were 5 μg of anti-ER clone F-10X (Santa Cruz Biotechnology) and 5 μg of anti–SRC-3 clone AX15.3 (Abcam, Cambridge, United Kingdom), and 5 μg of normal mouse IgG was used as a negative control (Santa Cruz Biotechnology). The washing of the magnetic beads used for the pulldowns were processed using Kingfisher Duo Prime magnetic particle processor (Thermo Scientific) according to the manufacturer’s instructions. The primers for the real-time PCR amplification of the *GREB1* proximal estrogen response element (ERE) enhancer site were 5′-GTG​GCA​ACT​GGG​TCA​TTC​TGA-3′ sense and 5′-CGA​CCC​ACA​GAA​ATG​AAA​AGG-3′ antisense (Integrated DNA Technologies). All treatments were performed in triplicate, the results represent the average of all replicates, and the error bars represent the S.D. in each treatment. Key differences are described in the *Results* section with 95% CIs.

#### Microarray Global Gene Analysis.

To assess the global gene transcription regulation over time in MCF-7:5C cells after treatment with the test compounds, we seeded the cells in six-well plates at a density of 300,000 cells per well. The next day after seeding the cells were treated with the indicated compounds for various durations, and the samples were harvested using TRIzol RNA Isolation Reagent (Invitrogen, Carlsbad, CA), and total RNA was isolated using RNeasy Mini kit (Qiagen, Hilden, Germany). The samples were processed and quality controlled at the University of Texas MD Anderson Cancer Center’s Sequencing and ncRNA core facility for analysis using Affymetrix human Clariom S microarrays (ThermoFisher Scientific). The raw data (CEL files) from the microarrays were quantified using Affymetrix Expression Console software. Each gene was then scaled by its average expression at time zero. A next-generation clustered heat map (Broom et al., 2017) was created using all genes showing a consistent change in expression over time and an average expression change of at least ± 5% after 96 hours (559 genes). Genes were clustered using clustered using correlation distance and Ward’s linkage. Cell lines were listed by time point and by treatment within each time point.

#### ERE DNA Pulldowns.

MCF-7:5C cells were grown in 25–15-cm Petri dishes in media containing charcoal stripped fetal bovine serum as indicated above. Nuclear extracts were then made and protein concentration determined as previously described for MCF-7 cells (Foulds et al., 2013). DNA pulldown assays used a doubly 5′-biotinylated 921 bp template containing four copies of the *Xenopus* vitellogenin ERE sequence immobilized onto Dynabeads M280 streptavidin as previously described (Foulds et al., 2013). One milligram of MCF7:5C nuclear extract and 0.5 μg recombinant ER*α* protein (Invitrogen) were added to 4×ERE-beads with either ethanol as vehicle control, 100 nM E_2_ or 1 μM of endoxifen, Z2OHTPE, 3OHTPE, or BPTPE for a 1.5-hour incubation at 4°C. Three washes were performed as previously described (Foulds et al., 2013), and the final coregulator-ER*α*-ERE DNA complexes were eluted from the beads in 30 μl 2× SDS sample buffer for mass spectrometry.

#### Mass Spectrometry.

Liquid chromatography–mass spectrometry (MS) was performed with label-free quantification, and the ERE/ER coregulator binding reactions were analyzed as previously described (Foulds et al., 2013). Briefly, the samples were minimally resolved on 10% NuPAGE gels, four broad-region bands were excised, and the proteins were in-gel digested with trypsin. For each experiment, the peptides were combined into two pools and measured on a Thermo Scientific Orbitrap Elite mass spectrometer coupled to an EASY nLC1200 UHPLC system. The raw data were searched in Proteome Discoverer suite with a Mascot 2.5 engine. The suite’s Peak Area Detector module was used for peptide quantification, and gpGrouper software was used for gene-centric inference and label-free quantitation based on the intensity-based absolute quantification method (Saltzman et al., 2018). All raw MS and gpGrouper result files have been deposited to the ProteomeXchange Consortium (http://proteomecentral.proteomexchange.org) in the MassIVE repository (MSV000082932) with the data set identifier PXD011052.

#### X-Ray Crystallography.

The 6×His-TEV–tagged ER-Y537S LBD mutant was expressed in *Escherichia coli* BL21 (DE3) and purified as previously described (Nettles et al., 2008; Speltz et al., 2016). LBD (5 mg/ml) was incubated with 1 mM compound and 1 mM glutamate receptor-interacting protein or SRC2-SP4 peptide (for 3OHTPE) at 4°C overnight. The LBD complexes were crystallized using hanging-drop vapor diffusion in 25% PEG 3350, 200 mM MgCl_2_, and 100 mM Tris pH 8.0. Clear rectangular crystals emerged between 2 and 5 days at room temperature. All crystals were cryoprotected in Paratone-N. Diffraction data were collected at the Advanced Photon Source SBC 19-BM beamline at 0.97 Å. Indexing, merging, and scaling were performed using HKL-3000 (Minor et al., 2006). Phenix was used for molecular replacement with Protein Data Bank entry 6CBZ used for the Z2OHTPE and BPTPE structures and 5DXE for 3OHTPE (Adams et al., 2010). Phenix was also used to generate ligand constraints. Refinement was conducted by iterative rounds of Phenix Refine and manual inspection using Coot (Emsley et al., 2010). Supplemental Table 1 shows data collection and refinement statistics. Final coordinates were deposited in the Protein Data Bank with the accession codes 6CZN, 6D0F, and 6D2A. The omit maps are shown in Supplemental Fig. 1.

#### Structure Preparation.

The experimental structures of ER*α* in complex with E_2_, Z2OHTPE, 3OHTPE, and BPTPE were used as starting points for molecular dynamics simulations. All structures were prepared for simulations using the Protein Preparation workflow implemented in Schrödinger 2019-1. In short, hydrogen atoms were added, bond and bond orders were assigned, water molecules beyond 5 Å of a heteroatom were deleted, and ionization states were generated at pH 7.4. Thus, Asp, Glu, Arg, and Lys residues were modeled as charged, and all Tyr residues were modeled as neutral. In each structure, the missing residues were modeled with Prime using as template the Protein Data Bank entry 1A52. These residues underwent special treatment during the minimization step of the solvation process. Finally, restrained minimizations of all atoms were performed, in default settings, until a root mean square deviation (RMSD) of 0.3 Å was reached. The wild-type structure of ER*α* was constructed by mutating Ser537 to Tyr and energetically minimizing residues within a range of 5 Å of Tyr while the remaining protein-ligand complex was kept frozen.

#### Molecular Dynamics Simulations.

The receptor-ligand complexes were solvated using the System Builder module of Desmond. Each complex was placed in a periodic orthorhombic water box, based on the TIP3P model, whose limits were set to 12 Å of protein atoms. Sodium and chloride ions were added to the systems to mimic the physiologic conditions (concentrations of 0.145 M) and to assure charge neutrality. To remove possible steric clashes due to the insertion of the missing residues and to relax the solvated systems, steepest descent energy minimizations were carried out using the Minimization module of Desmond. Positional constraints were applied to protein and ligands heavy atoms with a force constant of 0.5 kcal/(mol × Å2), excepting the initially missing atoms. All hydrogen atoms were allowed to move freely. Before performing the simulation runs, a series of minimizations and short molecular dynamics (MD) simulations were carried out to relax and equilibrate the systems using the default protocol of Desmond. Finally, all-atom MD simulations were performed starting from the equilibrated systems using Desmond implemented in Schrödinger 2019-1. The simulations were carried out at constant pressure (1 atm) and temperature (300 K), isothermal–isobaric ensemble ensemble, with default thermostat and barostat methods. Long-range electrostatic and van der Waals interactions were accounted for a distance cutoff of 10 Å, and no other restraints were applied. Each receptor-ligand system was simulated for 50 nanoseconds with a time step of 2 femtoseconds and a recording interval of coordinates of 2 picoseconds.

#### Trajectories Analysis.

Analysis of the computed trajectories was performed with the Simulation Integration Diagram module of Maestro 11.5. The RMSD and root mean square fluctuation (RMSF) of the receptor C*α* atoms relative to the reference structure were calculated. Trajectories were clustered to extract the most representative frames for each trajectory, in terms of the conformational space sampling. The clustering script from Desmond was used, the top 10 most populated clusters of each complex were retained, and the representative of each cluster was extracted. Then, binding free energies were computed using the molecular mechanics–generalized Born surface area method implemented in Schrödinger 2019-1. The H-bonds and hydrophobic contacts between ligands and key residues in the binding pocket, together with residues of helix 12 H-bonding to other residues, were monitored.

## Results

### 

#### Pharmacological Properties of Angular Estrogens in MCF-7:5C Cells.

**Fig. 2.**
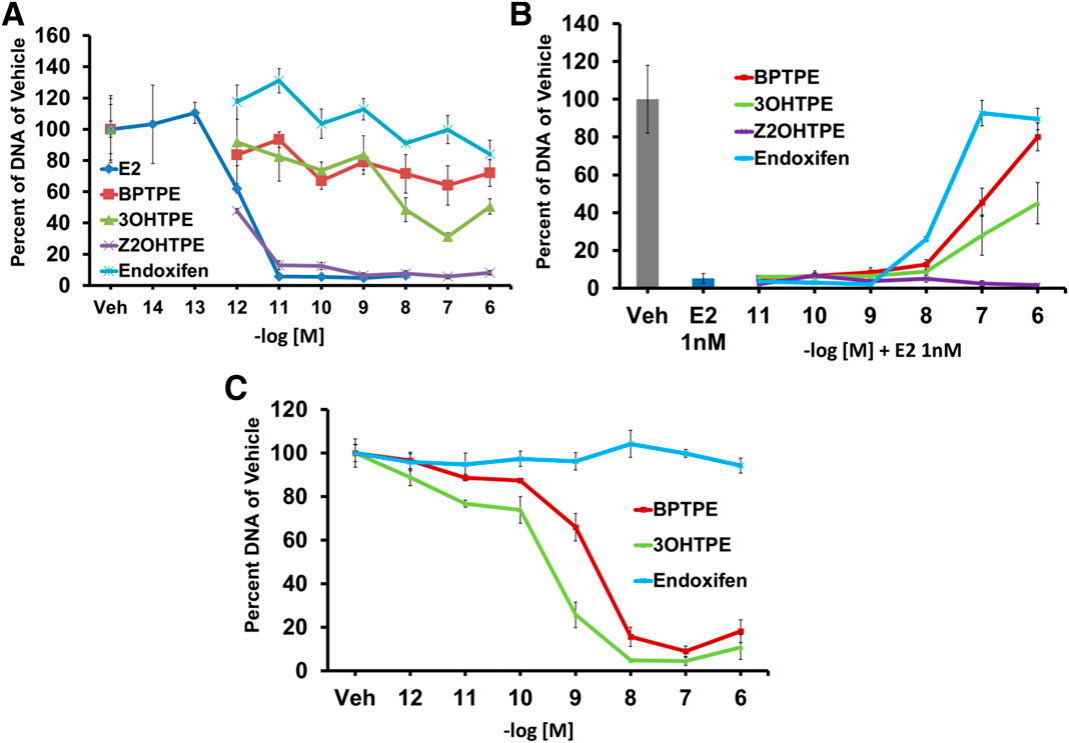
Cell proliferation assays in MCF-7:5C antihormone-resistant breast cancer cells. (A) Effects of test compounds alone after 7 days of treatment. Results show that the only two compounds able to completely inhibit the growth of the cells are E_2_ and Z2OHTPE, and the other two test compounds possess only minor inhibitory effects. Endoxifen was used as an antiestrogenic control and does not exhibit any biologic activity on the cells. (B) Antiestrogenic effects of test compounds in combination with 1 nM E_2_ after 7 days of treatment. Results show that all compounds, except Z2OHTPE, exhibit antiestrogenic effects after 7 days of treatment according to their intrinsic activity with the nonsteroidal antiestrogen endoxifen completely inhibiting the effect of E_2_. (C) Effects of test compounds alone on cells after 14 days of treatment. Results show that besides E_2_ and Z2OHTPE, the other two tested TPE derivatives BPTPE and 3OHTPE can inhibit the cell growth after 14 days of treatment. Endoxifen did not produce any inhibitory growth effect. All treatments were performed in triplicate; data represent the average of the replicates; error bars represent S.D.s with *n* = 3. Veh, vehicle.

To test the biologic properties of the angular estrogens BPTPE, 3OHTPE, and Z2OHTPE in MCF-7:5C cells, we used a DNA quantitation-based assay described in the *Experimental Procedures* section. Estrogenicity of these compounds in wild-type breast cancer cell line MCF-7 was previously described (Maximov et al., 2010). The planar estrogen E_2_ induced a reduction of live MCF-7:5C cells dose-dependently after 1 week of treatment (Fig. 2A). The lowest concentration that produced a partial reduction in cell DNA amount was 10^−12^ M E_2_ compared with vehicle control (95% CI 85.45–120.96 for vehicle and 95% CI 58.59–65.52 for 10^−12^ M E_2_) and a complete reduction of live cells at 10^−11^ M (95% CI, 4.63–7.00) (Fig. 2A). Angular estrogens BPTPE and 3OHTPE both only partially reduced the amount of live MCF-7:5C cells after 1 week of treatment, though both have induced dose-dependent effect with a maximum reduction of cells by an average of 30% for BPTPE and 50% for 3OHTPE at their highest concentrations of 10^−6^ M (95% CI 94.1–105.9 for BPTPE vehicle control and 95% CI 62.42–81.52 for 10^−6^ M BPTPE; 95% CI 77.66–122.34 for 3OHTPE vehicle control and 95% CI 44.97–56.04 for 10^−6^ M 3OHTPE) (Fig. 2A). Determination of IC_50_ for these compounds was inappropriate, since both are partial agonists, which is consistent with previous studies of BPTPE in MCF-7:5C cells after 7 days of treatment (Maximov et al., 2011; Obiorah and Jordan, 2014). Interestingly, Z2OHTPE, unlike other angular estrogens, demonstrated the same pharmacologic properties as E_2_, reducing the amount of live MCF-7:5C cells at tested concentrations of 10^−11^–10^−6^ M (95% CI 75.62–124.39 for Z2OHTPE vehicle control and 95% CI 9.79–16.32 for 10^−11^ M Z2OHTPE) (Fig. 2A). Endoxifen, a major biologically active metabolite of tamoxifen, was used as a triphenylethylene antiestrogenic control and did not induce any reduction of live cells compared with the vehicle control at any concentration point (Fig. 2A). This is also consistent with previously published results (Maximov et al., 2018). To test the antiestrogenic properties of the TPEs after 1 week of treatment, we treated MCF-7:5C cells with increasing concentrations of the compounds in combination with 1 nM E_2_. The results show that both BPTPE and 3OHTPE are able to inhibit 1 nM E_2_-induced apoptosis in cells according to their intrinsic activity alone at the highest tested concentrations of 10^−6^ M (Fig. 2B). However, Z2OHTPE was not able to inhibit E_2_-induced apoptosis at any tested concentrations, since it is a full agonist alone, like E_2_ (95% CI 2.27–5.41 for 1 nM Z2OHTPE and 95% CI 2.44–8.03 for 1 nM E_2_). Endoxifen was used as an antiestrogen control and was able to completely block estrogen-induced apoptosis in cells at top concentrations of 10^−7^ and 10^−6^ M with no difference in the number of live cells compared with vehicle control. Since it was demonstrated previously that both BPTPE (Obiorah and Jordan, 2014) and another TPE derivative EtOXTPE (Maximov et al., 2018) are able to dose-dependently induce apoptosis in MCF-7:5C cells after 2 weeks of treatment, we decided to test the effects of 3OHTPE and BPTPE on MCF-7:5C cells after 2 weeks of treatment. The results show that both TPEs are able to induce apoptosis in cells (Fig. 2C). Compounds BPTPE and 3OHTPE both reduce the amount of live MCF-7:5C cells dose-dependently and by more than 90% at a 10^−8^–10^−6^ M concentration range (95% CI 5.62–104.38 for BPTPE vehicle control vs. 95% CI 6.22–11.68 for 10^−8^ M BPTPE; 95% CI 92.7–103.7 for 3OHTPE vehicle control vs. 95% CI 4.23–5.6 for 10^−8^ M 3OHTPE), with 3OHTPE being more potent than BPTPE with an IC_50_ of 5 × 10^−10^ M compared with 5 × 10^−9^ M (Fig. 2C). Antiestrogen endoxifen did not change the number of viable cells at any concentration point (Fig. 2C).

#### Apoptotic Properties of Angular Estrogens in MCF-7:5C Cells.

**Fig. 3.**
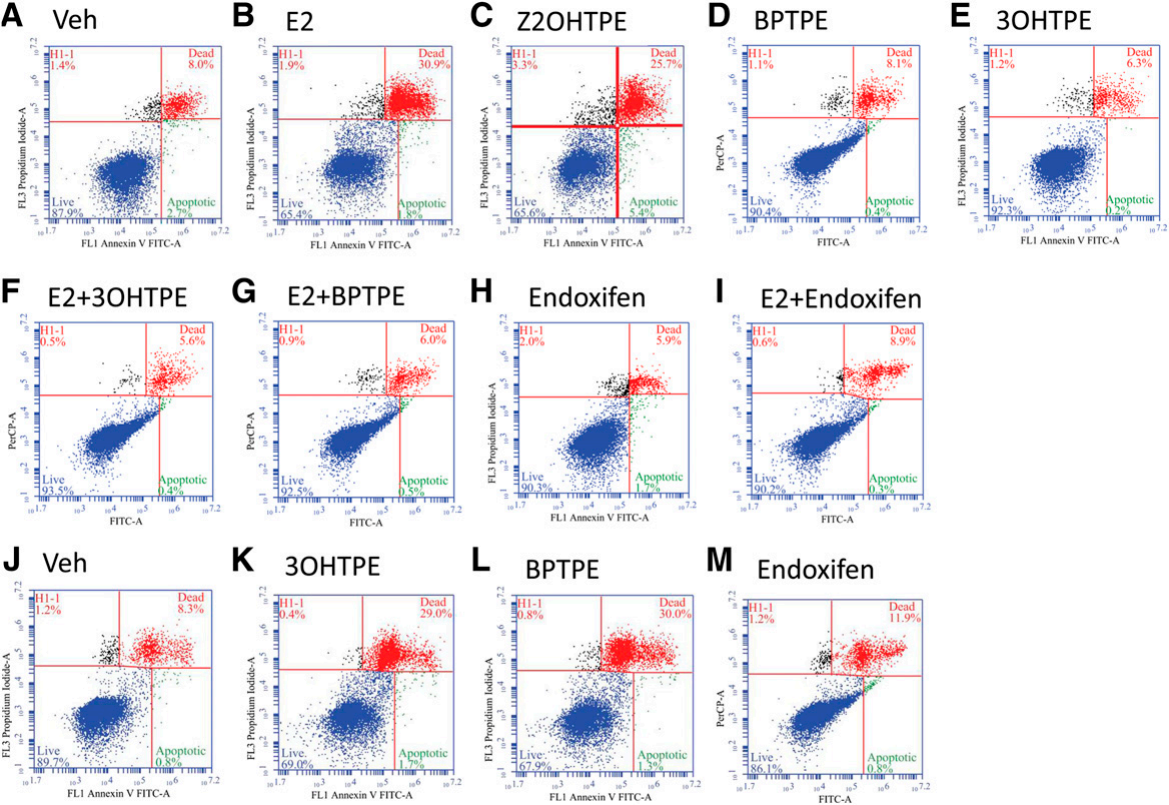
Annexin V staining of MCF-7:5C cells after 3-day treatments with vehicle (Veh) (A), 1 nM E_2_ (B), 1 μM Z2OHTPE (C), 1 μM BPTPE (D), 1 μM 3OHTPE (E), 1 μM 3OHTPE + 1 nM E_2_ (F), 1 μM BPTPE + 1 nM E_2_ (G), 1 μM endoxifen (H), and 1 μM endoxifen + 1 nM E_2_ (I), as well as 7 days of treatment with vehicle (J), 1 μM 3OHTPE (K), 1 μM BPTPE (L), and 1 μM endoxifen (M). The results demonstrate that E_2_ is able to induce positive Annexin V staining in MCF-7:5C cells after 3 days of treatment that can be blocked by the antiestrogen endoxifen, which does not have any effect at any time points tested. At the same time Z2OHTPE is the only angular estrogen able to produce positive Annexin V staining after 3 days of treatment. The other test compounds BPTPE and 3OHTPE were not able to produce similar levels of Annexin V staining until after 7 days of treatment and acting as antiestrogens, similar to endoxifen after 3 days of treatment, inhibiting E_2_-induced apoptosis. The panels represent one of the three experimental replicates; the quadrant lines were adjusted on the template in the Accuri C6 flow cytometer software to include all the cells in respective clusters based on the automatic gating parameters of the flow cytometer.

To determine the cause of the reduction of live cells after treatments with the test compounds we used Annexin V labeling with subsequent flow cytometry as described in the *Experimental Procedures* section. We treated the cells for 72 hours to detect apoptosis in cells with E_2_ treatment. Annexin V staining has indicated that there is an induction of apoptosis when compared with vehicle control (Fig. 3, A and B). Treatment with Z2OHTPE produced comparable percentage of apoptotic cells as E_2_ after 72 hours of treatment (Fig. 3, B and C). However, treatments with 3OHTPE and BPTPE did not produce any increase in the percentages of apoptotic cells after 72 hours when compared with vehicle control (Fig. 3, A, D, and E). Interestingly, compounds 3OHTPE and BPTPE were able to completely inhibit E_2_-induced apoptosis when combined with 1 nM E_2_ for 72 hours (Fig. 3, F and G). Endoxifen was used as an antiestrogenic control that did not produce any increase in the percentage of apoptotic cells. After 7 days of treatment, compounds 3OHTPE and BPTPE produced increase of the percentages of apoptotic cells to the similar extent as with E_2_ after 72 hours of treatment (Fig. 3, K and L). Endoxifen was used as an antiestrogenic control at both time points and produced no increase in the percentages of apoptotic cells compared with vehicle controls (Fig. 3, H and M) and also completely inhibited E_2_-induced apoptosis combined with 1 nM E_2_ (Fig. 3I) after 3 days of treatment.

**Fig. 4.**
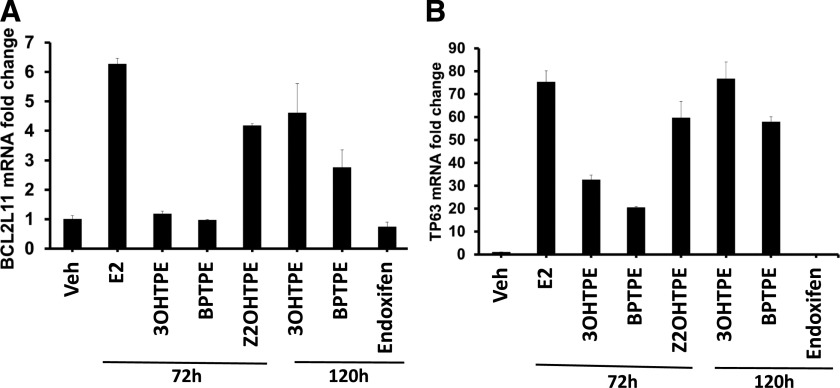
Modulation of the transcriptional activity of pro-apoptotic genes. (A) Effects on *BCL2L11* gene in MCF-7:5C cells after indicated durations with the indicated treatments. (B) Effects of the transcription of *TP63* gene after same treatments. Results show that compounds BPTPE and 3OHTPE require longer treatments to induce higher levels of mRNA transcription of pro-apoptotic genes compared with E_2_ after 72 hours of treatment, although still lower than E2. The only angular estrogen to induce apoptotic gene transcription at the same level after 72 hours of treatment as E2 is Z2OHTPE. All treatments were performed in triplicate; data represent the average of the replicates; error bars represent S.D.s with *n* = 3. Veh, vehicle.

We tested if the compounds induced the expression of proapoptotic genes, such as *TP63* and *BCL2L11* (Fig. 4). Estradiol induces proapoptotic gene *TP63* and *BCL2L11* transcription at a concentration of 1 nM after 72 hours of treatment (*TP63*: 95% CI 69.85–80.87 for E_2_ and 95% CI 0.83–1.18 for vehicle; *BCL2L11*: 95% CI 6.05–6.5 for 1 nM E2 and 95% CI 0.88–1.13 for vehicle) (Fig. 4). Compounds 3OHTPE and BPTPE do not induce transcription of *BCL2L11* after 72 hours of treatment (Fig. 4A) at 1 μM concentrations; however, *TP63* gene transcription was induced after the same duration of treatment, but less than E_2_ (95% CI 30.43–43.93 for 3OHTPE and 95% CI 20.02–21.03 for BPTPE) (Fig. 4B). Test compound Z2OHTPE induces transcription of *BCL2L11* gene after 72 hours of treatment (95% CI 4.1–4.25) but not at the same level as E_2_ (Fig. 4A). Compound Z2OHTPE activates transcription of *TP63* after 72 hours of treatment (95% CI 51.37–67.87) higher than BPTPE and 3OHTPE but lower than E_2_ (Fig. 4B). At longer durations of treatments both test compounds 3OHTPE and BPTPE were able to induce transcription of both *BCL2L11* and *TP63* genes (Fig. 4). Compounds 3OHTPE and BPTPE activated both genes after 120 hours of treatment (*TP63*: 95% CI 68.41–84.91 and 95% CI 55.2–60.43, respectively; *BCL2L11*: 95% CI 3.48–5.74 and 95% CI 2.1–3.42, respectively) (Fig. 4) but were less potent than E_2_ in induction of transcription of *BCL2L11* gene (Fig. 4A) and equivalent to Z2OHTPE (Fig. 4A), but were equivalent to E_2_ in *TP63* gene induction (Fig. 4B). Together with Annexin V flow cytometry data (Fig. 3) these data demonstrate that the reduction of live cells in the proliferation assays shown above (Fig. 2) is due to the induction of apoptosis in MCF-7:5C cells, which is delayed by 3OHTPE and BPTPE compared with E_2_ and Z2OHTPE.

#### Effects of Angular Estrogens on Global Gene Expression Profile in MCF-7:5C Cells.

**Fig. 5.**
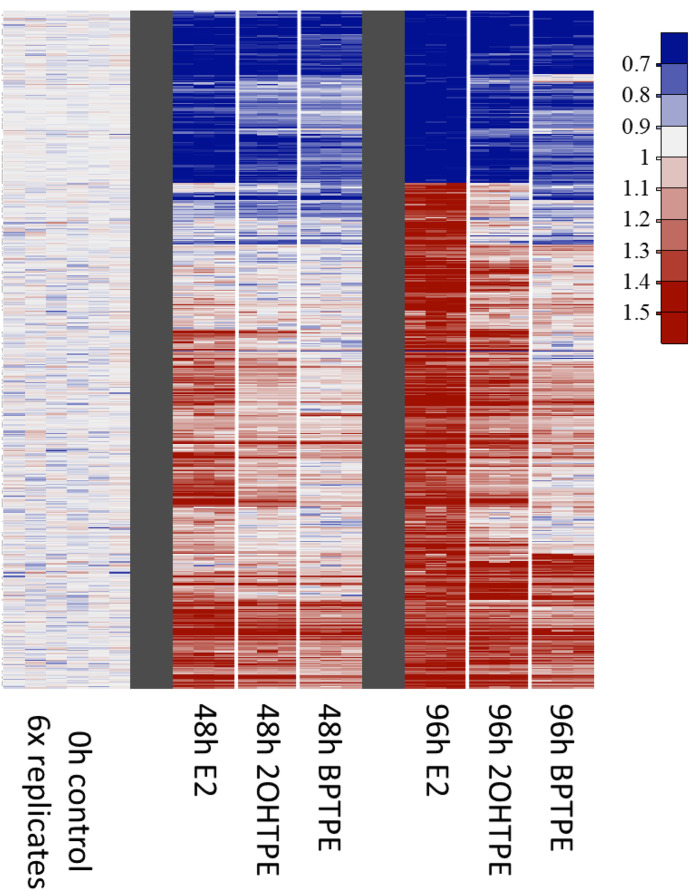
Modulation of the global gene expression profiles. The MCF-7:5C cells were treated with E2, Z2OHTPE, and BPTPE for indicated periods of time, and global gene transcriptional profiles were analyzed using microarrays as described in the *Experimental Procedures* section. Top up- and downregulated genes were selected based on the fold change at 96 hours vs. 0 hour control. Consistent with the biologic activities of the test compounds, E_2_ as full ER agonist is the most potent estrogen and induces transcription levels change of the majority of genes on the panel starting at 48 hours of treatment with further modulation of transcriptional level change in the gene panel at 96 hours of treatment. Compound Z2OHTPE induced a similar change as E_2_ at the same time points, however, less profoundly. Compound BPTPE was less potent than Z2OHTPE at the same time points in concordance with its partial agonist activity in the biologic assays. Overall, the global gene transcription profiles are consistent with the intrinsic biologic activity of the compounds.

Since Z2OHTPE demonstrated biologic effects and effects on the proapoptotic genes similar to E_2_, whereas the other TPEs showed partial agonist activities, we assessed the effect of Z2OHTPE and BPTPE on the overall gene expression (including genes not regulated by estrogens directly) compared with E_2_ in the MCF-7:5C cells. To assess the effects of the test compounds on the global transcriptional gene activity in MCF-7:5C cells, the cells were treated for 0, 48, and 96 hours with E_2_, Z2OHTPE, and BPTPE. We used the RNA from the treated cells for microarray analysis, and the regulation of some of the up- and downregulated genes with the highest fold changes was analyzed as described in the *Experimental Procedures* section. The results indicate that, compared with 0 hours control, E_2_ considerably up- and downregulated numerous genes (Fig. 5) after both 48 and 96 hours of treatment. Compared with the effect of E_2_ on transcriptional activity of these genes, test compound Z2OHTPE was able to regulate the same genes in a similar fashion as E_2_ at both time points but, compared with 0 hours control, was less effective than E_2_ (Fig. 5). At the same time, compound BPTPE was also able to considerably change the expression of the same panel of genes when compared with the 0 hour control; however, it was much less effective than both E_2_ and Z2OHTPE in regulating the expression of these genes with a certain cluster of genes actually being downregulated even after 96 hours of treatment compared with 0 hour control. This is the opposite of the effects of E_2_ and Z2OHTPE (Fig. 5). These results are not only consistent with BPTPE being a partial agonist and the least potent test compound, as shown in our biologic assays and in the analysis on the apoptosis-related genes described above, but also demonstrate that Z2OHTPE is similar to E_2_ compared with BPTPE. Patterns are consistent. The full list of genes included in the heatmap analysis is presented in the Supplemental File 1. To further study the effects of the test compounds on gene transcription, we used quantitative real-time PCR to measure the expression of select estrogen-regulated genes that are directly dependent on the transcriptional activity of the ligand-bound ER.

#### Effects of Test Compounds on Transcriptional Activity of the ER.

**Fig. 6.**
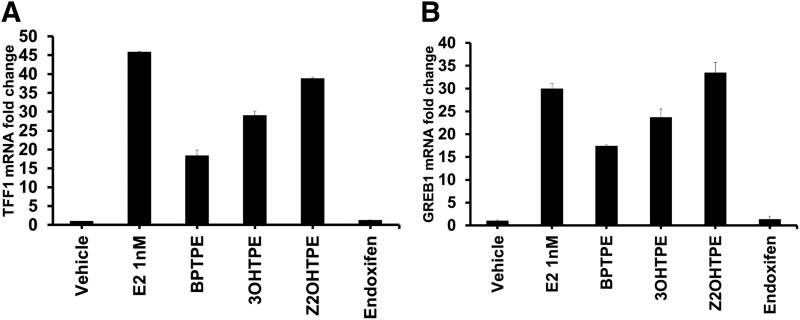
Modulation of the transcriptional activity of estrogen-responsive genes. (A) Effects on well known estrogen-responsive gene *TFF1* mRNA expression in MCF-7:5C cells after 24 hours of treatment with E_2_ at 1 nM concentration and 1 µM for other test compounds. The results show that the tested compounds induce *TFF1* gene mRNA expression partially and lower than the levels induced by E_2_, all in concordance with their biologic activity. (B) *GREB1* mRNA expression after 24 hours of treatment with E_2_ at 1 nM concentration and 1 µM for other test compounds. The results are very similar to results seen in *TFF1* gene regulation, with only Z2OHTPE being a full agonist. All treatments were performed in triplicate; data represent the average of the replicates; error bars represent S.D.s with *n* = 3.

To assess the transcriptional activity of the ER on estrogen-responsive genes, MCF-7:5C cells were treated with test compounds, and quantitative real-time PCR was performed as described in the *Experimental Procedures* section. The estrogen-responsive genes selected for evaluation were *TFF1* and *GREB1* (Fig. 6). Treatments were performed for 24 hours in triplicate. The results show that E_2_ was able to increase the levels of *TFF1* and *GREB1* mRNAs compared with vehicle controls (*TFF1*: 95% CI 45.65–46.03 for E_2_ and 95% CI 0.96–1.05 for vehicle control; *GREB1*: 95% CI 28.69–31.24 for E_2_ and 95% CI 0.9–1.12 for vehicle control) (Fig. 6). All TPE derivatives tested produced an increase in *TFF1* and *GREB1* mRNAs but with some differences (*TFF1*: 95% CI 27.79–30.27 for 3OHTPE, 95% CI 16.88–19.96 for BPTPE and 95% CI 38.44–39.22 for Z2OHTPE; *GREB1*: 95% CI 21.57–25.78 for 3OHTPE, 95% CI 17.09–17.71 for BPTPE, and 95% CI 30.85–36.05 for Z2OHTPE). Compounds BPTPE and 3OHTPE produced only partial effect, less than E_2_, with BPTPE being less potent than 3OHTPE. Interestingly, Z2OHTPE produced a full agonist effect comparable to E_2_ for induction of both genes (Fig. 6). The antiestrogen endoxifen did not increase transcriptional activity for either of the genes evaluated when compared with vehicle controls (Fig. 6). These results are consistent with the biologic activity of the test TPEs in the previous experiments, where compound Z2OHTPE acted as a full agonist like E_2_ while BPTPE and 3OHTPE acted as partial agonists, with BPTPE being the least potent TPE. Overall, these results show that the positioning of the hydroxyl groups on the ligands gives compounds different potency when compared with each other.

#### Recruitment of ER and Its Major Coactivator SRC-3 to the *GREB1* Gene.

**Fig. 7.**
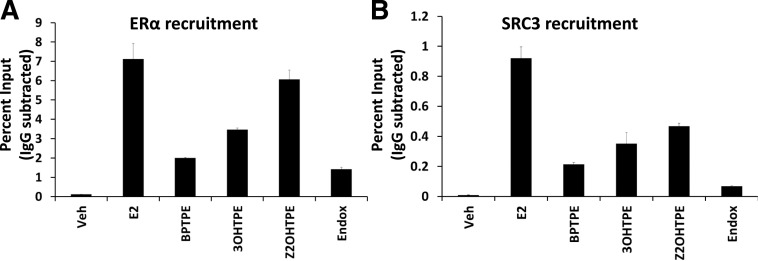
ChIP assay showing recruitment of ER (A) and SRC-3 (B) at *GREB1* proximal estrogen-responsive element after a 45-minute treatment with test compounds in MCF7:5C cells at 1 nM concentration for E_2_ and 1 µM for other test compounds. (A) The results show that all compounds were able to recruit ER to the *GREB1* ERE, with Z2OHTPE recruiting approximately the same levels as E_2_, a full agonist, with no statistical difference between the two and higher levels than BPTPE and 3OHTPE. Both BPTPE and 3OHTPE were able to recruit ER only partially when compared with E_2_, with BPTPE being the least potent of the tested TPEs. (B) At the same time all test TPEs did not recruit SRC-3 coactivator to the *GREB1* gene at the same levels as E_2_, however, higher than vehicle control. Compound Z2OHTPE recruited the most SRC-3 compared with other two TPEs. All treatments were performed in triplicate; data represent the average of the replicates; error bars represent S.D.s with *n* = 3. Endox, endoxifen; veh, vehicle.

To test the differential recruitment of the ER and the SRC-3 coactivator to this target gene, we performed chromatin immunoprecipitation (ChIP) assays in MCF-7:5C cells treated with the tested compounds. The ChIP assays were performed as described in the *Experimental Procedures* section. The results show very strong recruitment of the ER to the *GREB1* proximal ERE enhancer site after the treatment of MCF-7:5C cells with E_2_ (Fig. 7A). The average levels of the ER recruitment after treatment with two of the TPEs were lower than E_2_; however, Z2OHTPE was comparable to E_2_ (95% CI 5.52–6.61 for Z2OHTPE and 95% CI 6.19–8.04 for E_2_) (Fig. 7A). The recruitment of the ER with the tested TPEs correlated with their respective biologic activity in the cells. Treatment of cells with BPTPE conferred the least ER ERE occupancy, 3OHTPE increased the ER occupancy, and Z2OHTPE revealed the highest ER chromatin binding (Fig. 7A). Treatment of cells with all TPEs resulted in higher ER ERE occupancy than in vehicle control treated cells (95% CI 1.97–2.03 for BPTPE, 95% CI 3.35–3.56 for 3OHTPE, and 95% CI 0.11–0.12 for vehicle) (Fig. 7A). Endoxifen was used as an antiestrogen control, and cells treated with it displayed less ER recruited to the ERE as compared with all the TPEs, but still at a level higher than the vehicle control (95% CI 1.31–1.54 for endoxifen) (Fig. 7A). SRC-3 is a major ER coactivator in breast cancer cells (Anzick et al., 1997), and this is why its recruitment to the *GREB1* proximal ERE enhancer site was assayed. SRC-3 coactivator recruitment was highest with E_2_ treatment (Fig. 7B) and all the TPEs recruited less SRC-3 (95% CI 0.008–0.01 for vehicle control, 95% CI 0.84–1.01 for vehicle control, 95% CI 0.84–1.01 for E_2_, 95% CI 0.2–0.23 for BPTPE, 95% CI 0.27–0.43 for 3OHTPE, and 95% CI 0.44–0.49 for Z2OHTPE) (Fig. 7B). Endoxifen recruited more SRC-3 than in the vehicle treatment but less than any tested estrogenic compound (95% CI 0.06–0.07) (Fig. 7B).

#### Effects of Test Compounds on Coregulator Recruitment to DNA-Bound ER.

**Fig. 8.**
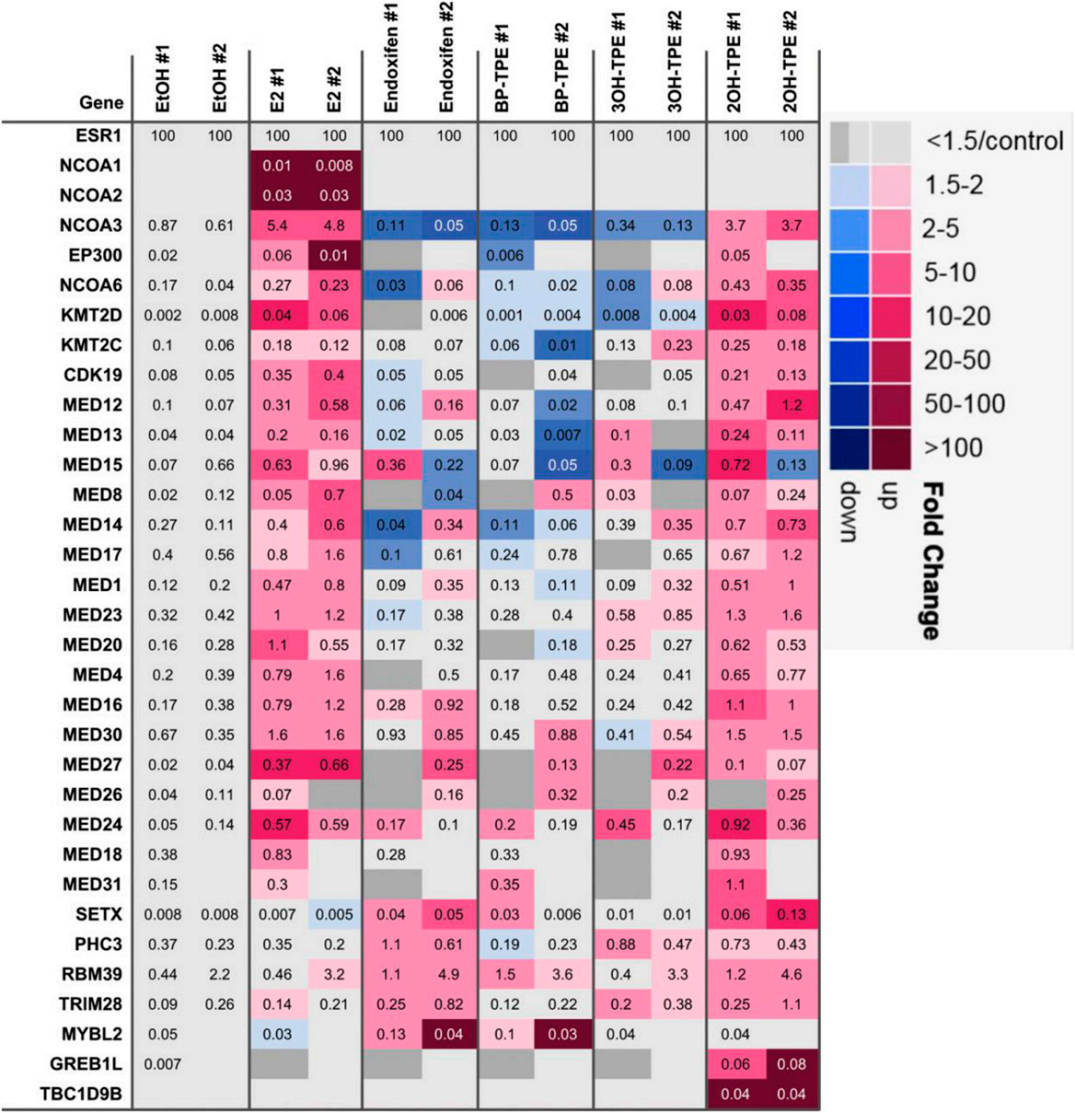
Differential recruitment of coregulators from MCF7:5C cells to DNA-bound recombinant ER in the presence of different ligands. Duplicate cell-free ERE DNA pulldown reactions were performed for MCF7:5C cell lysates treated with vehicle control (EtOH) and five different ligands (100 nM E_2_, 1 µM of endoxifen, BPTPE, 3OHTPE, or Z2OHTPE). Bound proteins were quantified with label-free mass spectrometry using intensity-based absolute quantification expression values from gpGrouper algorithm (Saltzman et al., 2018). All protein quantities were further normalized by and scaled to the ER amount (set as 100%). Coregulator enrichment is depicted as a row-normalized heatmap for enhanced (pink to purple color) or diminished (light to dark blue color) binding in different ligands, as compared with corresponding vehicle control for each replicate set (#1 or #2). For cases where fold change calculations resulted in infinite decrease due to sporadic missing identifications, darker gray was used to represent indecision. Official gene symbols are shown on the leftmost column. Note that NCOA1–3, NCOA6, EP300, mediator subunits (MEDs), and KMT2C/KMT2D were previously defined as E_2_-enriched coactivators (Foulds et al., 2013; Gates et al., 2018).

Since we observed significant differences in ER-target gene transcription and ER and SRC-3 occupancy of EREs promoted by the different TPEs, we next characterized, in an unbiased manner, coregulators recruited to ER bound to EREs in the presence of different TPE ligands, using E_2_ and endoxifen as positive and negative controls for coactivator binding. To do this, we performed duplicate cell-free ERE DNA pulldown assays (Foulds et al., 2013) with recombinant ER, nuclear extract made from MCF-7:5C cells, and different ER ligands. After coregulator complexes were formed and washed, bound proteins were identified by liquid chromatography–MS. As expected (Foulds et al., 2013; Gates et al., 2018), E_2_ recruited known coactivators such as p160/steroid receptor coactivator (SRC) family members (NCOA1–3), NCOA6, p300 (EP300), and the Mediator complex (see MED subunits), in addition to KMT2C/2D histone methyltransferases (Fig. 8; Supplemental File 2). Endoxifen, as expected, did not recruit these coactivators but instead recruited other coregulators such as SETX, PHC3, RBM39, TRIM28, and MYBL2. The recruitment of TRIM28 (also called KAP1), which has potent corepressor activity (Iyengar and Farnham, 2011), is consistent with the effect of endoxifen on ER target genes. Also consistent with the above effects of ER-target genes (Fig. 6), we found that Z2OHTPE recruited the E_2_-enriched coactivators (except for NCOA1 and NCOA2) as an agonist ligand but additionally recruited the endoxifen-enriched coregulators (except for MYBL2) and two more “unique” coregulators GREB1L and TBC1D9B (Fig. 8). The partial agonists BPTPE and 3OHTPE did not recruit many of the E_2_-enriched coactivators and only a subset of endoxifen-enriched coregulators (e.g., only RBM39 and MYBL2 with BPTPE; PHC3 and TRIM28 with 3OHTPE). In sum, our biochemical assays strongly suggest that the differential transcriptional potency defined above for the three TPEs in ER-expressing cells stems from the collective recruitment patterns of E_2_-enriched coactivators (or lack thereof) and endoxifen-enriched coregulators, with Z2OHTPE, but not other TPEs, recruiting the vast majority of E_2_-enriched coactivators.

#### X-Ray Structure Analysis.

**Fig. 9.**
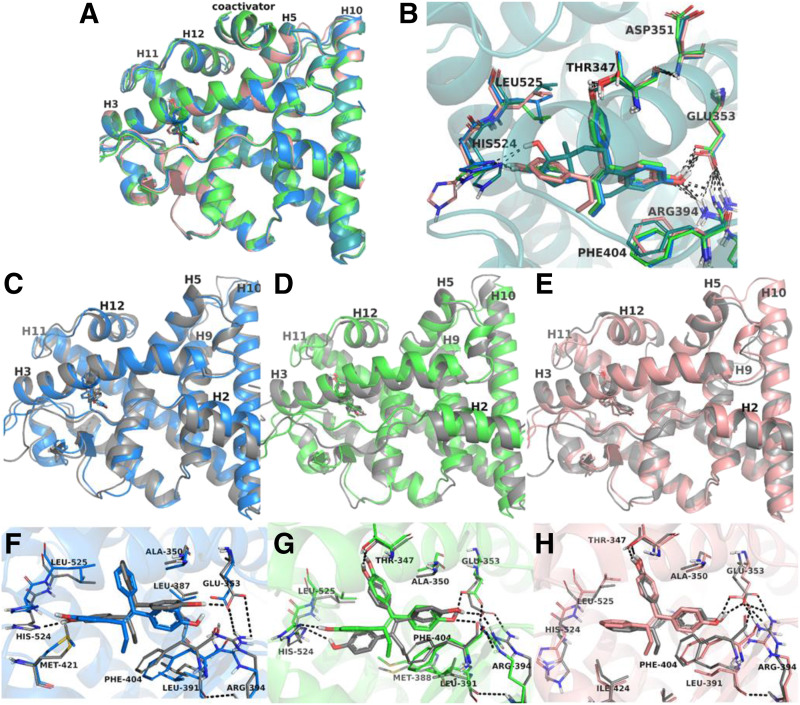
Molecular dynamics simulations of the wild-type ER LBD with TPE derivatives. Experimental structures of ER-LBD co-crystallized with E_2_ (teal), Z2OHTPE (blue), 3OHTPE (green), and BPTPE (pink) are superimposed (A), and the contacts between the ligands and critical amino acids of the binding site are shown (B). For each ligand-receptor complex snapshots taken from the MD trajectory (colored in gray) are overlaid with their experimental structures (C–E, same color code as in A). Close views of the ER binding pocket with Z2OHTPE (F), 3OHTPE (G), and BPTPE (H) show small variations between the experimental structures and the representative conformations extracted from the MD simulations. The same color code is used in pictures (C–H); MD snapshots are colored in gray, whereas the experimental structures are depicted in blue for Z2OHTPE, green for 3OHTPE, and pink for BPTPE. The black dashed lines show the H-bonds between ligands and the amino acids of the binding site.

Analysis of the experimental structures of ER*α* Tyr537Ser complexed with the TPE derivatives Z2OHTPE, 3OHTPE, and BPTPE showed similar conformations of the receptors with a high degree of similarity to the ER*α* Tyr537S-E_2_ complex. All X-ray structures adopt the canonical agonist conformation with helix 12 positioned over the binding site, sealing ligands inside. No major differences have been noticed in the binding modes of the ligands and positioning of helix 12 between TPE complexes and the reference structure, ER*α* Tyr537S-E_2_ (Fig. 9A). In the binding pocket, all ligands recapitulate to some extent the H-bond network specific to E_2_. Thus, the common H-bonding to Glu353 and Arg394 via a phenolic hydroxyl is shared by all ligands. The additional phenolic hydroxyl of Z2OHTPE and 3OHTPE forms H-bonds with His524, like E_2_, whereas a feature specific to 3OHTPE and BPTPE is the formation of an H-bond with Thr347 (Fig. 9B). The hydrophobic interactions account for the remaining contacts with the binding pocket. From Fig. 9, A and B, one can see that the differences between structures are minor using this technology, and the features responsible for the observed biologic behavior could not be identified. Thus, we performed MD simulations for TPE derivatives bound to wild-type ER*α* LBD to investigate the conformational dynamics of ligands binding and their influence upon the receptor conformation. Additionally, experimental X-ray structures were obtained for the mutant Tyr537Ser ER*α*-LBD and with a coactivator peptide glutamate receptor-interacting protein 1 that was not recruited by the TPEs ER*α* complexes in the biologic experiments performed the wild-type ER*α* (see above section) and was not included in the molecular dynamics simulations. Furthermore, the mutated residue, Ser537, is next to helix 12 and interacts with Asp351, which in turn connects to Thr347, a residue involved in H-bond formation with 3OHTPE and BPTPE (Fig. 9B). Thus, it can be expected that mutation Tyr537Ser could influence the position of essential residues interacting with the ligands. For these reasons, wild-type ER*α*-ligand complexes without coactivators were built from the experimental structures and used in 50-nanosecond MD simulation for each system.

#### Structural Analysis of MD Simulations.

To explore the conformational stability of the simulations and ensure that all models have reached equilibrium, RMSDs of the proteins’ C*α* atoms relative to their position in the first frame were monitored for each trajectory. The calculated RMSD values indicate the complexes reaching stable states after approximately 5 nanoseconds (Supplemental Fig. 1; Supplemental Table 2). The sole exception is BPTPE, which fluctuates more and reaches the plateau around 12 nanoseconds (Supplemental Fig. 1). Also, RMSD has been calculated for the C*α* atoms of helix 12. The results show helix 12 fluctuating similarly as the native proteins, with slightly lower values of RMSD for BPTPE (Supplemental Table 2). Next, the stability of the ligands relative to the protein and its binding site, together with the internal fluctuations of ligands atoms, was investigated (Supplemental Table 2). The analysis shows small internal fluctuations of the ligands, around 0.4 Å, and the ligands bound stable in the binding sites, with RMSD values ranging from 1.04 ± 0.4 Å for E_2_ up to 1.43 ± 0.21 Å for Z2OHTPE (Supplemental Table 2).

The RMSF of the residues was monitored along the trajectories to investigate the mobility of the protein chain and the dynamic features in ligand binding. Comparing the RMSF calculated for C*α* atoms of all simulated systems, we noticed that the most significant fluctuations overlap with flexible domains in the protein; e.g., the most prominent peak identified is located between residues 456 and 469 (Supplemental Fig. 2). These residues are part of a loop connecting H9 and H10, a flexible domain in the receptor, and part of H10 (residues 466–469), which is not involved in ligand binding and does not interact with H12. BPTPE displays the most substantial fluctuations in this peak and explains the larger values observed for RMSD. The second peak lies between residues 330 and 340, matching to the loop connecting H2 and H3. Another region of high mobility, mainly for BPTPE, corresponds to the peak located between residues 370 and 377. These residues belong partially to the loop connecting H4 and H5, extending to the N-terminal end of H5, part of the coactivator binding site. In the MD simulations performed for the systems with a coactivator, this region does not show increased flexibility because it is occupied by the coactivator protein (data not shown). In the coactivator free systems, the domain is open, exposed toward the solvent, explaining the increased flexibility. Supplemental Fig. 2 displays the RMSF values plotted per residue for each system, together with the experimental B-factors. By comparing these parameters, it is evident that RMSF values correlate with B-factors; the flexibility regions parallel parts of the proteins of high B-factor values. In the following, we describe the structural features that discriminate between ligands and explain the observed biologic profile for the wild-type form of ER*α*.

#### Analysis of Protein-Ligand Interactions in the Modeled Wild-Type ER*α* Systems.

To understand the features responsible for the observed biologic behavior of TPE derivatives, we have analyzed their interactions with the active site residues of ER*α*. The X-ray structures of ER*α* with the ligands show the presence of a conserved H-bonds network involving the hydroxyl groups of the ligands and the side chains of His524, Glu353, Arg394, and a water molecule (Fig. 9, F–H). Additionally, 3OHTPE and BPTPE H-bond to the hydroxyl group of Thr347 (Fig. 9, G and H). These contacts, together with the hydrophobic interactions, were monitored for all trajectories and are summarized in Supplemental Fig. 2 as timeline representations, whereas the frequencies of occurrence are presented in Supplemental Table 3. Similar to E_2_, the H-bond to His524 occurs over 95% of the time for Z2OHTPE and 3OHTPE, whereas the interaction with Glu353 occurs with lower frequency, in the following order: Z2OHTPE (75%), 3OHTPE (66%), and BPTPE (65%). A direct H-bond to Arg394 is not observed in the simulations, only via a water bridge, with frequencies below 20% (Supplemental Table 3). A distinctive feature is the H-bond between the hydroxyl group of Thr347 and the phenolic hydroxyl of 3OHTPE and BPTPE (Fig. 9, G and H), which occurs over 90% of the trajectories, indicating stable interactions with this residue, confirmed by low RMSF values of the residue (Supplemental Fig. 3, A and B). The hydrophobic contacts, mainly with residues Met343, Ala 350, Met388, Leu384, Leu391, Leu428, Leu525, and pi-pi stacking interactions with Phe404, define the remaining contacts between the ligands and the receptor. Interestingly, the experimental B-factors show high values for His524 and Leu525 in the structures of Z2OHTPE and 3OHTPE, but not for E_2_, indicating increased flexibility of the residues in these structures. However, the RMSF of His524 and Leu525 calculated based on C*α* atoms and side chains show minimal fluctuations, similar to the RMSF values of the residues in the E_2_ complex (Supplemental Fig. 3, A–C). These findings show that His524 is stabilized through H-bonding to Z2OHTPE and 3OHTPE inducing stability in the binding pocket, reinforced by the hydrophobic contacts with Leu525. Additional information on RMSF calculations are included in the Supplemental Fig. 4. Information on the timeline of the interactions of amino acid residues in the LBD with the test compounds within the 50-nm simulation time can be viewed in the Supplemental Fig. 5.

To select the most representative structure out of the conformational space sampled in each MD simulation, the trajectories were clustered, and the top 10 most populated clusters were retrieved for each trajectory. Then, molecular mechanics–generalized Born surface area calculations were performed to select the most appropriate receptor-ligand complex in each cluster based on the binding energy and the overall energy of the system. The comparison of the most representative conformations with the native ER*α*-TPEs structures has highlighted the common features, as well as those which differentiate among them (Fig. 9, C–H). All modeled structure recapitulate the known binding modes, with minor differences in the orientation of the ligands and orientation of some amino acids. Thus, Arg394 side chain shifts slightly toward Glu353 to form ionic interactions (Fig. 9, F–H). Differences have been noticed in the overall structures of the proteins when compared with the native structures. Helices 3 and 11 harbor parts of the binding site and are slightly displaced in the modeled structures. The displacement propagates to helix 12 and the coactivator binding site, mainly helices 3 and 5 (Fig. 9, C–E). Of the three TPE ligands, the most notable displacement of H12 has been noticed in the ER*α*-BPTPE complex, with an average RMSD of 2 Å relative to the reference structure (Fig. 9E), followed by 3OHTPE and Z2OHTPE with average RMSD of 1.3 Å (Fig. 9, C and D). The less stable binding of BPTPE, which is mainly due to the missing interaction with His524 and less favorable hydrophobic contacts, could be causing the drift of H12 (Fig. 9H).

To gain more information about the elements that could potentially differentiate between structures, the interaction between Asn348 (H3) and Tyr537 (H11) was monitored because of the close vicinity with Thr347, involved in stable H-bond with 3OHTPE and BPTPE (Fig. 9, G and H). Thr347 is next to Asn348, which H-bonds to Tyr537 (70% of the time in E_2_ and Z2OHTPE complexes but in lower frequencies in BPTPE and 3OHTPE structures). The orientation of the Thr347 side chain is shifted in 3OHTPE and BPTPE complexes so that the hydroxyl group is drawn closer to the ligand. The methyl group is oriented in the pocket delignated by Leu536, Tyr537, and Leu540, entering in steric clashes with the side chain of Leu536. This orientation, together with the proximity of the ligand hydroxyl group, pushes the residues Leu536, Tyr537, and Leu540, adding instability to the systems and probably allowing the displacement of helix 12 to a slightly different position. In Z2OHTPE complex the same orientation has been seen, but the phenolic hydroxyl is missing, and Thr347 is not drawn to the ligand. The H-bond between Asn348 and Tyr537 is not affected, showing frequencies similar to the E_2_ complex (roughly 70% of the simulation time).

## Discussion

It has been previously reported (Song et al., 2001; Lewis et al., 2005a,b) that E_2_ can trigger apoptosis in antiestrogen-resistant breast cancer cells. This in vitro model has clinical relevance since low- and high-dose estrogen treatments have antitumor actions in LTED breast cancer (Jordan, 2014; Coelingh Bennink et al., 2017). Here we expand knowledge about the structure-function relationship of nonsteroidal estrogens (Fig. 1) and estrogen-induced apoptosis in LTED breast cancer cell line MCF-7:5C. For the first time, we demonstrate that a TPE derivative with an unsubstituted phenyl ring Z2OHTPE reduces the number of viable MCF-7:5C cells at the same rate as E_2_ (Fig. 2) via apoptosis, as shown by Annexin V (Fig. 3) and induction of proapoptotic genes (Fig. 4). This compound is a full agonist as demonstrated by the global gene expression profile (Fig. 5) as well as estrogen-responsive gene expression regulation (Fig. 6). Compound Z2OHTPE was demonstrated as closest to E_2_ gene expression profile starting at 48 hours, a previously demonstrated (Obiorah et al., 2014) time point of irreversible apoptosis induction in MCF-7:5C cells. At the same time, compounds BPTPE and 3OHTPE have a delay in apoptosis induction and act as antiestrogens in the first week of treatment (Figs. 2 and 3). These two compounds are partial agonists as shown by global gene expression profile (Fig. 5 for BPTPE) and estrogen-responsive gene transcription regulation (Fig. 6).

All these data are consistent with previously described partial agonist biology for compound EtOXTPE (Maximov et al., 2018), BPTPE, and 3OHTPE (Obiorah and Jordan, 2014; Obiorah et al., 2014), which demonstrated a delayed apoptotic profile in the same MCF-7:5C cells. This differential apoptotic activity between the test compounds can be linked to conformational differences in the LBD of the ER (Fig. 9) that in turn can affect the transcriptional activity of the receptor.

The transcriptional activity of the ER is dependent on the recruitment of coregulators (O’Malley, 2004). It is a well established fact that ligands can induce different conformations of the ER, which is necessary for the ER transactivation (Beekman et al., 1993). The LXXLL motif is a crucial surface site for the recruitment of coactivators to the ER liganded with an agonist upon the conformational change of the LBD of the ER and its external surface (Heery et al., 1997; Chang et al., 1999) as well as the stability of the receptor (Wijayaratne and McDonnell, 2001). On the other hand, antiestrogens can produce various conformational changes that will affect the stability of the ER protein (Wijayaratne and McDonnell, 2001) or promote the recruitment of corepressors (Huang et al., 2002). Here we have confirmed and advanced previous studies (Beekman et al., 1993; Chang et al., 1999; Wijayaratne and McDonnell, 2001; Huang et al., 2002) using ERE DNA pulldowns with MS to study coregulator binding as well as ChIP assays. The results of the ChIP assays show only partial recruitment of a well known coactivator of the ER SRC-3 with all the test TPEs compared with E_2_ (Fig. 7). However, we also demonstrate partial recruitment of the ER protein itself to the *GREB1* gene proximal enhancer region with BPTPE and 3OHTPE (Fig. 7). At the same time, compound Z2OHTPE is able to recruit a comparable amount of the ER protein to the same genomic DNA region as E_2_ (Fig. 7). Since we have demonstrated that Z2OHTPE is able to induce transcriptional activity of the *GREB1* gene as well as E_2_ after 24 hours of treatment, but with only partial recruitment of SRC-3 coactivator, we used ERE DNA pulldowns with subsequent MS identification of all ER coregulators recruited to the ER bound with the test compounds (Fig. 8).

Here, for the first time, we demonstrate the differential recruitment of coregulators to the ER liganded with the test compounds (Fig. 8). We demonstrate that Z2OHTPE recruits less SRC-3 (labeled as NCOA3) than E_2_ consistent with the ChIP results; however, the complex is able to recruit the same suite of other coregulators as E_2_. Most importantly, the Z2OHTPE complex also recruited a subset of endoxifen-enhanced coregulators, including the TRIM28 corepressor (Fig. 8). At the same time, BPTPE and 3OHTPE, consistent with their profiles of their transcriptional activity modulation of estrogen-regulated gene, recruited less coactivators and more corepressors, which are also recruited with the antiestrogenic control endoxifen (Fig. 8). Together, these data are consistent with the biologic profiles of the compounds in the MCF-7:5C cells (Sengupta et al., 2013; Maximov et al., 2014; Obiorah and Jordan, 2014) and suggest that each of the test TPEs produces a different conformational change in the ER LBD, which in turn regulates the recruitment of coregulators and its transcriptional activity based on their structures.

Since the biologic activity of a TPE estrogen is dependent on the conformation of the ER LBD, as previously reported with EtOXTPE (Maximov et al., 2018), we performed X-ray crystallography of the ER LBD in complex with the test compounds. Estradiol induces complete closure of helix 12 over the LBD (Fig. 9). Paradoxically, all of our current test TPEs cause closure of the LBD with helix 12 as well, locking the ligand inside (Fig. 9). However, a Tyr537Ser mutant ER LBD, which was used for crystallography purposes, enhances agonist conformation of the LBD and may be an artifact that does not occur in wild-type ER. The X-ray crystallography technology for the wild-type ER LBD is not available to our team. As a result, we performed cumulative 200-nanosecond (50 nanoseconds for each investigated system) classic MD simulations against ER wild type in complex with E_2_, Z2OHTPE, 3OHTPE, and BPTPE to investigate the dynamics of binding for these ligands and their influence upon receptor conformation. The trajectory analysis revealed equilibrated simulations and identified regions of the receptors prone to flexibility, which correlate with the experimental B-factors. By comparing the flexibility of these regions for TPE derivatives and E_2_, no differences were identified that could account for the observed biologic behavior. Next, contacts and interactions between the ligands and protein were monitored to highlight common and different features among ligands. Z2OHTPE and 3OHTPE recapitulate the conserved H-bond network found in the agonist ER-E_2_ system, with H-bond to H524 kept stable for almost the whole simulation time, whereas the H-bond to Glu353 is found less frequently than for E_2_. The later indicates a less stable agonist conformation of the wild-type ER-TPEs complex, compared with ER-E_2_. The H-bond of 3OHTPE and BPTPE to Thr347 is found roughly 90% of the simulation time, indicating a stable contact.

We were able to differentiate residue Thr347 as the amino acid that is displaced by both BPTPE and 3OHTPE that, in turn, is able to produce steric hindrance with residues Leu536, Tyr537, and Leu540, as demonstrated by molecular dynamics. At the same time neither E_2_ nor Z2OHTPE causes the shift of Thr347, which, subsequently, allows residues Asn348 and Tyr537 to form an H-bond for approximately 70% of the simulation time. These results indicate that the differences in the orientation and interaction of specific amino acid residues in the LBD bound with the hydroxyl groups of BPTPE and 3OHTPE predetermine the differential pharmacology observed in vitro. These results contrast with the X-ray crystallography for the EtOXTPE compound (Maximov et al., 2018), where we have observed a different orientation of the H12 on the ER LBD bound with EtOXTPE. However, the large ethoxy group on EtOXTPE creates steric hindrance with H12, similar to endoxifen (Maximov et al., 2018). These data demonstrate a novel structure-function relationship of angular TPE derived estrogens and the ER.

In summary, the closure of helix 12 of the ER LBD bound with a ligand promotes the recruitment of the coregulators to the ER to form a transcriptionally active complex. We have observed differences in recruitment of coregulators between the test compounds (Figs. 7 and 8) that segregate into patterns related to biology (see *Results* section). We have demonstrated, using ChIP assays, that the test TPEs recruit the ER protein and the SRC-3 coactivator to an ERE in correlation with their biologic activity. Compound Z2OHTPE recruited almost as much ER protein to the *GREB1* proximal ERE enhancer site as E_2_; however, 3OHTPE and BPTPE recruited less ER protein, and all test TPEs recruited less SRC-3 compared with the levels observed for the ER-E_2_ complex.

Overall, these data support the hypothesis that the alterations in the positioning of the hydroxyl groups on the TPE derivatives tested result in the specific shifts of Thr347 with both BPTPE and 3OHTPE, which is not the case with Z2OHTPE. This, in turn, leads to the production of unique conformations of the ER LBD as demonstrated with molecular dynamics modeling. These novel conformations of the ER LBD, when compared with E_2_, result in a differential recruitment of the SRC-3 coactivator and multiple other types of coregulator molecules. One in particular is the corepressor TRIM28, which also binds to the antiestrogen endoxifen-ER complex (Fig. 8). The different ER complexes define the partial agonist activity of the test TPEs on the transcription of estrogen responsive genes. Most importantly, the cluster of novel coregulator molecules in the partial agonist complexes (BPTPE and 3OHTPE) potentially explains the delayed induction of estrogen-induced apoptosis in LTED breast cancer cells.

The central role of the ER-ligand complex in the modulation of the life and death of breast cancer cells is programmed by these studies of molecular modulation. Future molecular studies with novel compounds used to trigger estrogen-induced apoptosis in clinical studies (O’Regan et al., 2018; Schmidt et al., 2019) will focus on the spectrum of molecular coregulators recruited to trigger early apoptosis.

## Abbreviations

BPTPE: bisphenoltriphenylethylene
ChIP: chromatin immunoprecipitation
CI: confidence interval
DES: diethylstilbestrol
E_2_: 17*β*-estradiol
ER: estrogen receptor
ERE: estrogen response element
EtOXTPE: ethoxytriphenylethylene
LBD: ligand-binding domain
LTED: long-term estrogen deprived
MD: molecular dynamics
MS: mass spectrometry
3OHTPE: trihydroxytriphenylethylene
PCR: polymerase chain reaction
RMSD: root mean square deviation
RMSF: root mean square fluctuation
TPE: triphenylethylene
Z2OHTPE: Z-isomer of dihydroxytriphenylethylene

## References

[B1] Adams PD Afonine PV Bunkóczi G Chen VB Davis IW Echols N Headd JJ Hung LW Kapral GJ Grosse-Kunstleve RW (2010) PHENIX: a comprehensive Python-based system for macromolecular structure solution. Acta Crystallogr D Biol Crystallogr 66:213–221. 20124702 10.1107/S0907444909052925 PMC2815670

[B2] Anderson GL Limacher M Assaf AR Bassford T Beresford SA Black H Bonds D Brunner R Brzyski R Caan B Women’s Health Initiative Steering Committee (2004) Effects of conjugated equine estrogen in postmenopausal women with hysterectomy: the Women’s Health Initiative randomized controlled trial. JAMA 291:1701–1712. 15082697 10.1001/jama.291.14.1701

[B3] Anderson WF Katki HA Rosenberg PS (2011) Incidence of breast cancer in the United States: current and future trends. J Natl Cancer Inst 103:1397–1402. 21753181 10.1093/jnci/djr257 PMC3176776

[B4] Anzick SL Kononen J Walker RL Azorsa DO Tanner MM Guan XY Sauter G Kallioniemi OP Trent JM Meltzer PS (1997) AIB1, a steroid receptor coactivator amplified in breast and ovarian cancer. Science 277:965–968. 9252329 10.1126/science.277.5328.965

[B5] Ariazi EA Cunliffe HE Lewis-Wambi JS Slifker MJ Willis AL Ramos P Tapia C Kim HR Yerrum S Sharma CG (2011) Estrogen induces apoptosis in estrogen deprivation-resistant breast cancer through stress responses as identified by global gene expression across time. Proc Natl Acad Sci USA 108:18879–18886. 22011582 10.1073/pnas.1115188108 PMC3223472

[B6] Beekman JM Allan GF Tsai SY Tsai MJ O’Malley BW (1993) Transcriptional activation by the estrogen receptor requires a conformational change in the ligand binding domain. Mol Endocrinol 7:1266–1274. 8264659 10.1210/mend.7.10.8264659

[B7] Broom BM Ryan MC Brown RE Ikeda F Stucky M Kane DW Melott J Wakefield C Casasent TD Akbani R (2017) A galaxy implementation of next-generation clustered heatmaps for interactive exploration of molecular profiling data. Cancer Res 77:e23–e26. 29092932 10.1158/0008-5472.CAN-17-0318 PMC5715806

[B8] Chang C Norris JD Grøn H Paige LA Hamilton PT Kenan DJ Fowlkes D McDonnell DP (1999) Dissection of the LXXLL nuclear receptor-coactivator interaction motif using combinatorial peptide libraries: discovery of peptide antagonists of estrogen receptors alpha and beta. Mol Cell Biol 19:8226–8239. 10567548 10.1128/mcb.19.12.8226 PMC84907

[B9] Chlebowski RT Anderson GL Aragaki AK Manson JE Stefanick M Pan K Barrington W Kuller LH Simon MS Lane D (2019) Long-term influence of estrogen plus progestin and estrogen alone use on breast cancer incidence: The Women’s Health Initiative randomized trials, in *San Antonio Breast Cancer Symposium*; San Antomio, TX. Abstract #GS5-00.

[B10] Coelingh Bennink HJ Verhoeven C Dutman AE Thijssen J (2017) The use of high-dose estrogens for the treatment of breast cancer. Maturitas 95:11–23. 27889048 10.1016/j.maturitas.2016.10.010

[B11] Ellis MJ Gao F Dehdashti F Jeffe DB Marcom PK Carey LA Dickler MN Silverman P Fleming GF Kommareddy A (2009) Lower-dose vs high-dose oral estradiol therapy of hormone receptor-positive, aromatase inhibitor-resistant advanced breast cancer: a phase 2 randomized study. JAMA 302:774–780. 19690310 10.1001/jama.2009.1204 PMC3460383

[B12] Emsley P Lohkamp B Scott WG Cowtan K (2010) Features and development of Coot. Acta Crystallogr D Biol Crystallogr 66:486–501. 20383002 10.1107/S0907444910007493 PMC2852313

[B13] Foulds CE Feng Q Ding C Bailey S Hunsaker TL Malovannaya A Hamilton RA Gates LA Zhang Z Li C (2013) Proteomic analysis of coregulators bound to ERα on DNA and nucleosomes reveals coregulator dynamics. Mol Cell 51:185–199. 23850489 10.1016/j.molcel.2013.06.007 PMC3900250

[B14] Gates LA Gu G Chen Y Rohira AD Lei JT Hamilton RA Yu Y Lonard DM Wang J Wang SP (2018) Proteomic profiling identifies key coactivators utilized by mutant ERα proteins as potential new therapeutic targets. Oncogene 37:4581–4598. 29748621 10.1038/s41388-018-0284-2 PMC6095836

[B15] Gottardis MM Jordan VC (1988) Development of tamoxifen-stimulated growth of MCF-7 tumors in athymic mice after long-term antiestrogen administration. Cancer Res 48:5183–5187. 3409244

[B16] Gottardis MM Wagner RJ Borden EC Jordan VC (1989) Differential ability of antiestrogens to stimulate breast cancer cell (MCF-7) growth in vivo and in vitro. Cancer Res 49:4765–4769. 2758410

[B17] Haddow A (1970) David A. Karnofsky memorial lecture. Thoughts on chemical therapy. Cancer 26:737–754. 4918638 10.1002/1097-0142(197010)26:4<737::aid-cncr2820260402>3.0.co;2-t

[B18] Haddow A Watkinson JM Paterson E Koller PC (1944) Influence of synthetic oestrogens on advanced malignant disease. BMJ 2:393–398. 20785660 10.1136/bmj.2.4368.393 PMC2286289

[B19] Heery DM Kalkhoven E Hoare S Parker MG (1997) A signature motif in transcriptional co-activators mediates binding to nuclear receptors. Nature 387:733–736. 9192902 10.1038/42750

[B20] Huang HJ Norris JD McDonnell DP (2002) Identification of a negative regulatory surface within estrogen receptor alpha provides evidence in support of a role for corepressors in regulating cellular responses to agonists and antagonists. Mol Endocrinol 16:1778–1792. 12145334 10.1210/me.2002-0089

[B21] Iyengar S Farnham PJ (2011) KAP1 protein: an enigmatic master regulator of the genome. J Biol Chem 286:26267–26276. 21652716 10.1074/jbc.R111.252569 PMC3143589

[B22] Jordan VC (2003) Tamoxifen: a most unlikely pioneering medicine. Nat Rev Drug Discov 2:205–213. 12612646 10.1038/nrd1031

[B23] Jordan VC (2014) Linking estrogen-induced apoptosis with decreases in mortality following long-term adjuvant tamoxifen therapy. J Natl Cancer Inst 106:dju296. 25269699 10.1093/jnci/dju296 PMC4271028

[B24] Jordan VC Schafer JM Levenson AS Liu H Pease KM Simons LA Zapf JW (2001) Molecular classification of estrogens. Cancer Res 61:6619–6623. 11559523

[B25] Kennedy BJ (1965) Hormone therapy for advanced breast cancer. Cancer 18:1551–1557. 5845796 10.1002/1097-0142(196512)18:12<1551::aid-cncr2820181206>3.0.co;2-1

[B26] LaCroix AZ Chlebowski RT Manson JE Aragaki AK Johnson KC Martin L Margolis KL Stefanick ML Brzyski R Curb JD WHI Investigators (2011) Health outcomes after stopping conjugated equine estrogens among postmenopausal women with prior hysterectomy: a randomized controlled trial. JAMA 305:1305–1314. 21467283 10.1001/jama.2011.382 PMC3656722

[B27] Lewis JS Meeke K Osipo C Ross EA Kidawi N Li T Bell E Chandel NS Jordan VC (2005a) Intrinsic mechanism of estradiol-induced apoptosis in breast cancer cells resistant to estrogen deprivation. J Natl Cancer Inst 97:1746–1759. 16333030 10.1093/jnci/dji400

[B28] Lewis JS Osipo C Meeke K Jordan VC (2005b) Estrogen-induced apoptosis in a breast cancer model resistant to long-term estrogen withdrawal. J Steroid Biochem Mol Biol 94:131–141. 15862958 10.1016/j.jsbmb.2004.12.032

[B29] Lønning PE Taylor PD Anker G Iddon J Wie L Jørgensen LM Mella O Howell A (2001) High-dose estrogen treatment in postmenopausal breast cancer patients heavily exposed to endocrine therapy. Breast Cancer Res Treat 67:111–116. 11519859 10.1023/a:1010619225209

[B30] Maximov P Sengupta S Lewis-Wambi JS Kim HR Curpan RF Jordan VC (2011) The conformation of the estrogen receptor directs estrogen-induced apoptosis in breast cancer: a hypothesis. Horm Mol Biol Clin Investig 5:27–34. 10.1515/HMBCI.2010.047 PMC3109984 21660224

[B31] Maximov PY Abderrahman B Fanning SW Sengupta S Fan P Curpan RF Rincon DMQ Greenland JA Rajan SS Greene GL (2018) Endoxifen, 4-hydroxytamoxifen and an estrogenic derivative modulate estrogen receptor complex mediated apoptosis in breast cancer. Mol Pharmacol 94:812–822. 29739819 10.1124/mol.117.111385 PMC6022805

[B32] Maximov PY Fernandes DJ McDaniel RE Myers CB Curpan RF Jordan VC (2014) Influence of the length and positioning of the antiestrogenic side chain of endoxifen and 4-hydroxytamoxifen on gene activation and growth of estrogen receptor positive cancer cells. J Med Chem 57:4569–4583. 24805199 10.1021/jm500569h PMC4059272

[B33] Maximov PY Myers CB Curpan RF Lewis-Wambi JS Jordan VC (2010) Structure-function relationships of estrogenic triphenylethylenes related to endoxifen and 4-hydroxytamoxifen. J Med Chem 53:3273–3283. 20334368 10.1021/jm901907u PMC2867604

[B34] Minor W Cymborowski M Otwinowski Z Chruszcz M (2006) HKL-3000: the integration of data reduction and structure solution--from diffraction images to an initial model in minutes. Acta Crystallogr D Biol Crystallogr 62:859–866. 16855301 10.1107/S0907444906019949

[B35] Nettles KW Bruning JB Gil G Nowak J Sharma SK Hahm JB Kulp K Hochberg RB Zhou H Katzenellenbogen JA (2008) NFkappaB selectivity of estrogen receptor ligands revealed by comparative crystallographic analyses. Nat Chem Biol 4:241–247. 18344977 10.1038/nchembio.76 PMC2659626

[B36] Obiorah I Sengupta S Curpan R Jordan VC (2014) Defining the conformation of the estrogen receptor complex that controls estrogen-induced apoptosis in breast cancer. Mol Pharmacol 85:789–799. 24608856 10.1124/mol.113.089250 PMC3990021

[B37] Obiorah IE Jordan VC (2014) Differences in the rate of oestrogen-induced apoptosis in breast cancer by oestradiol and the triphenylethylene bisphenol. Br J Pharmacol 171:4062–4072. 24819221 10.1111/bph.12762 PMC4243979

[B38] O’Malley BW (2004) Results of a search for the mechanisms of steroid receptor regulation of gene expression. Ann N Y Acad Sci 1038:80–87. 15838100 10.1196/annals.1315.014

[B39] O’Regan R Hurley R Sachdev JC Tonetti DA Thatcher GR Venuti RP Dudek AZ (2018) Study TTC-352-101: phase 1 study of TTC-352 in patients with metastatic breast cancer (BC) progressing on endocrine therapy. J Clin Oncol 36:TPS1108. 10.1007/s10549-020-05787-z 32696319

[B40] Saltzman AB Leng M Bhatt B Singh P Chan DW Dobrolecki L Chandrasekaran H Choi JM Jain A Jung SY (2018) gpGrouper: a peptide grouping algorithm for gene-centric inference and quantitation of bottom-up Proteomics data. Mol Cell Proteomics 17:2270–2283. 30093420 10.1074/mcp.TIR118.000850 PMC6210220

[B41] Schmidt M Hönig A Verhoeven C Almstedt K Battista M Lenhard HG Krijgh J Coelingh Bennink H (2019) Estetrol for treatment of advanced ER+ breast cancer [abstract], in *Proceedings of the 2018 San Antonio Breast Cancer Symposium*; 2018 Dec 4–8; San Antonio, TX. *Cancer Res* 79(4 Suppl): Abstract nr P4-13-12.

[B42] Sengupta S Obiorah I Maximov PY Curpan R Jordan VC (2013) Molecular mechanism of action of bisphenol and bisphenol A mediated by oestrogen receptor alpha in growth and apoptosis of breast cancer cells. Br J Pharmacol 169:167–178. 23373633 10.1111/bph.12122 PMC3632247

[B43] Siegel RL Miller KD Jemal A (2015) Cancer statistics, 2015. CA Cancer J Clin 65:5–29. 25559415 10.3322/caac.21254

[B44] Song RX Mor G Naftolin F McPherson RA Song J Zhang Z Yue W Wang J Santen RJ (2001) Effect of long-term estrogen deprivation on apoptotic responses of breast cancer cells to 17beta-estradiol. J Natl Cancer Inst 93:1714–1723. 11717332 10.1093/jnci/93.22.1714

[B45] Speltz TE Fanning SW Mayne CG Fowler C Tajkhorshid E Greene GL Moore TW (2016) Stapled peptides with γ-methylated hydrocarbon chains for the estrogen receptor/coactivator interaction. Angew Chem Int Ed Engl 55:4252–4255. 26928945 10.1002/anie.201510557 PMC4964982

[B46] Wijayaratne AL McDonnell DP (2001) The human estrogen receptor-alpha is a ubiquitinated protein whose stability is affected differentially by agonists, antagonists, and selective estrogen receptor modulators. J Biol Chem 276:35684–35692. 11473106 10.1074/jbc.M101097200

[B47] Wolf DM Jordan VC (1993) A laboratory model to explain the survival advantage observed in patients taking adjuvant tamoxifen therapy. Recent Results Cancer Res 127:23–33. 8502820 10.1007/978-3-642-84745-5_4

[B48] Yao K Lee ES Bentrem DJ England G Schafer JI O’Regan RM Jordan VC (2000) Antitumor action of physiological estradiol on tamoxifen-stimulated breast tumors grown in athymic mice. Clin Cancer Res 6:2028–2036. 10815929

